# The outcome of possible events following the hospital discharge of patients with HFmrEF after MI as compared with those with HFmrEF without MI: a propensity score matching analysis

**DOI:** 10.3389/fcvm.2025.1622220

**Published:** 2025-11-17

**Authors:** Zhican Liu, Lingling Zhang, Jianping Zeng, Mingyan Jiang

**Affiliations:** 1 Department of Pulmonary and Critical Care Medicine, Xiangtan Central Hospital, The Affiliated Hospital of Hunan University, Xiangtan, China; 2 Medical Department, Xiangtan Central Hospital, The Affiliated Hospital of Hunan University, Xiangtan, China; 3 Department of Cardiology, Xiangtan Central Hospital, The Affiliated Hospital of Hunan University, Xiangtan, China

**Keywords:** heart failure with mildly reduced ejection fraction, myocardial infarction, percutaneous coronary intervention, outcome, PSMA

## Abstract

**Background:**

Clinical studies on heart failure (HF) with mildly reduced left ventricular ejection fraction (HFmrEF) are gradually increasing. However, relatively few studies have examined patients with HFmrEF after myocardial infarction (MI), and the prognosis of such patients remains unclear. Therefore, we conducted a retrospective evaluation of HFmrEF patients with/without MI using a propensity score matching analysis (PSMA).

**Methods:**

A total of 1,691 patients with HFmrEF were included in this study. Of these patients, 873 had a diagnosis of MI, and 818 did not. After propensity score matching, we used Kaplan–Meier analysis and Cox regression to compare all-cause mortality, cardiovascular death, or HF readmission (CV events).

**Results:**

After the first PSMA, the MI group had a lower risk of all-cause mortality [hazard ratio (HR) 0.6; 95% confidence interval (95% CI) 0.5–0.8] compared with the non-MI group; however, there was no significant difference in the incidence of CV events (HR 0.9; 95% CI 0.7–1.2). After the second PSMA, which additionally matched for PCI performance in the MI group, there were no differences in the risk of all-cause mortality (HR 1.0; 95% CI 0.7–1.5) or CV events (HR 1.1; 95% CI 0.8–1.5) between the MI and non-MI groups.

**Conclusions:**

There was no difference in all-cause mortality and CV events between patients with HFmrEF with and without MI. However, among patients with HFmrEF and MI, those who underwent PCI had a much lower risk of all-cause mortality compared with patients with HFmrEF without MI and those with HFmrEF after MI who did not undergo PCI.

## Introduction

Patients with heart failure (HF) and a left ventricular ejection fraction (LVEF) between the ranges for heart failure with reduced ejection fraction (HFrEF) and heart failure with preserved ejection fraction (HFpEF) are referred to as “HF with mid-range ejection fraction (EF)” or “HF with mildly reduced EF” (1). Because LVEF is lower than normal, they are classiﬁed as having HF with mildly reduced EF(HFmrEF) according to the 2022 American Heart Association/American College of Cardiology/Heart Failure Society of America Guideline for the Management of Heart Failure (2). In addition, the 2021 European Society of Cardiology heart failure guidelines define HFmrEF as HF with LVEF 41%–49% (3). In recent years, the global incidence of heart failure seems to have progressively increased each year (4–6). One of the main reasons for the increase in HF is the substantial increase in the survival rate following a diagnosis of MI, which inadvertently affects the survival of more patients with left ventricular dysfunction. Although the number of studies reported on patients with HFmrEF has been increasing, few have focused on patients with HFmrEF after MI. These patients may have a different prognosis than other patients with HF. Thus, we conducted a retrospective evaluation to compare outcomes between HFmrEF patients with and without a diagnosis of MI.

## Patients and methodologies

The study protocol was approved by the Ethics Committee of Xiangtan Central Hospital (Xiangtan, China) and conformed to the principles outlined in the Declaration of Helsinki (7). Informed consent was obtained from all patients or their guardians before the inception of the study protocols.

This study included patients admitted to our hospital between 1 January 2015 and 31 August 2020. HFmrEF was defined according to the ESC 2021 guidelines as a LVEF of 41%–49% measured by transthoracic echocardiography during the index hospitalization, combined with symptoms and/or signs of heart failure corresponding to New York Heart Association (NYHA) functional class II–IV. Myocardial infarction (MI) was diagnosed according to the Fourth Universal Definition of MI. In this study, all MI cases occurred prior to or during the index hospitalization in which HFmrEF was diagnosed. Patients with a history of MI after the diagnosis of HFmrEF were not included. The temporal sequence was determined based on hospital admission records, discharge summaries, and prior medical documentation.

A total of 1,691 patients with HFmrEF were included in the study: 873 were diagnosed with MI, and 818 did not suffer from MI. Malignant tumors or other non-cardiac diseases with an expected survival time of less than 1 year were excluded from both groups. The primary endpoint of this study was all-cause mortality, and the secondary endpoints were cardiovascular (CV) events, defined as a composite of cardiovascular death and readmission for heart failure.

### Data collection and follow-up

Demographic and procedural data were collected from hospital charts or databases. Follow-up was conducted on all study participants until 31 August 2021, through clinical telephone interviews and community visits. The median follow-up time was 33 months (interquartile range: 20–50 months).

### Statistical analysis

Continuous variables are expressed as the mean ± standard deviation. The first propensity score matching analysis (PSMA) was performed using a multivariate logistic regression model based on the following factors: age, sex, body mass index (BMI), diabetes, hypertension, hyperlipidemia, current smoker, coronary heart disease, atrial fibrillation, previous stroke, chronic obstructive pulmoriary disease, renal insufficiency, creatinine, New York Heart Association functional class, use of respirator, and use of electrocardiogram monitoring. Pairs of patients with or without MI were derived within a quarter of the standard deviation of the estimated propensity using 1:1 greedy nearest-neighbor matching. This strategy resulted in 439 matching pairs per group [percutaneous coronary intervention (PCI) was not included as a matching variable in the first PSMA]. The second propensity score matching analysis was performed, adding the factor of whether PCI had been performed while retaining other factors from the first analysis. This yielded 308 pairs per group.

The propensity score matching analyses were intentionally structured to reflect the study's primary objective—namely, to explore prognostic differences between HFmrEF patients with and without MI and to further assess the effect of PCI within the MI subgroup. Alternative grouping strategies, such as dividing patients according to primary or secondary outcomes, may provide additional perspectives but would shift the analytic framework away from MI status, which was the central hypothesis of this study. Moreover, by definition, patients without MI did not undergo PCI, and this limitation has been acknowledged in the Discussion section.

Clinical characteristics between groups were compared using *t*-tests for continuous measures and chi-square tests for categorical variables. The Kaplan–Meier method was used to estimate cumulative event incidence, and a Cox proportional hazards model was constructed to assess the hazard ratio (HR) for each event between the two groups. Cox regression was conducted as a univariable analysis because the PSM procedure had already balanced all measured covariates, making further multivariable adjustment unnecessary. The balance of measured variables between groups after propensity score matching was analyzed using paired *t*-tests for continuous measures and McNemar's test for categorical variables. After propensity score matching, differences in cumulative event rates were analyzed using the stratified Cox procedure.

*P*-values were obtained using the Kruskal–Wallis rank-sum test for continuous variables and Fisher's exact probability test for count variables. Results were considered significant when *P* < 0.05. All analyses were performed with R (http://www.R-project.org) and EmpowerStats software (https://www.empowerstats.com, X&Y Solutions, Inc., Boston, MA, USA).

## Results

**Figure 1:**
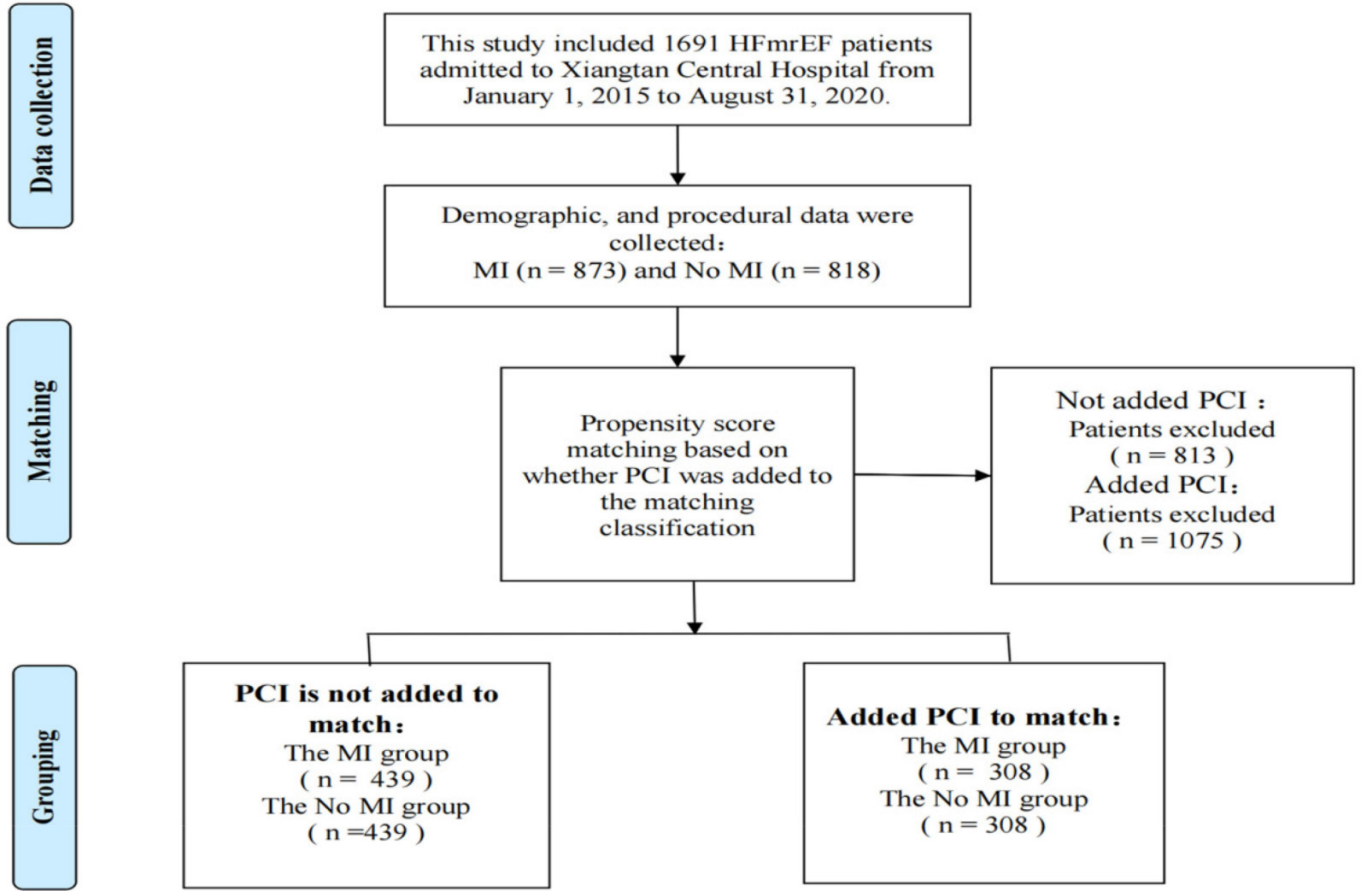
Flow diagram for participant screening, eligibility, and analysis.

Table 1 shows the baseline characteristics of outcome events before propensity score matching (*N* = 1,691). Risk factors for all-cause mortality were: age [HR 1.1; 95% confidence interval (95% CI) 1.0–1.1; *P* < 0.001], hypertension (HR 1.5; 95% CI 1.2–1.9; *P* < 0.001), atrial fibrillation (HR 1.7; 95% CI 1.3–2.2; *P* < 0.001), diabetes (HR 1.3; 95% CI 1.0–1.6; *P* = 0.033), previous stroke (HR 2.1; 95% CI 1.5–2.8), *P* < 0.001), chronic obstructive pulmonary disease (COPD) (HR 2.3; 95% CI 1.7–3.1; *P* < 0.001), renal insufficiency (HR 2.2; 95% CI 1.7–2.8; *P* < 0.001), NYHA class III (HR 1.6; 95% CI 1.2–2.0; *P* < 0.001), NYHA class IV (HR 2.1; 95% CI 1.6–2.8; *P* < 0.001), ventilator use (HR 3.5; 95% CI 1.9–6.3; *P* < 0.001), and creatinine ≥106 µmol/L (HR 2.7; 95% CI 2.2–3.4; *P* < 0.001). The presence or absence of myocardial infarction was a protective factor for all-cause death (HR 0.5; 95% CI 0.4–0.7; *P* < 0.001). PCI or not is a protective factor for all-cause death (HR 0.3; 95% CI 0.2–0.4; *P* < 0.001) and cardiovascular events (HR 0.7; 95% CI 0.6–0.9; *P* = 0.001). Therefore, these factors that had a significant impact on the outcome events and other common influencing factors were included in the propensity score matching analysis. The purpose of performing two propensity score matching analyses was to make the two matched groups more comparable and determine whether the protective factors of myocardial infarction on outcome events were related to PCI. Among the 1,691 HFmrEF patients enrolled, 873 had been diagnosed with an MI, whereas 818 had reported being free of any episodes of an MI. A total of 439 matching pairs were obtained after the first propensity score matching analysis, and 308 matching pairs were obtained after the second propensity score matching analysis (Figure 1).

**Table 1:** Baseline characteristics of outcome events before propensity score matching.

Variable	Total (*N* = 1,691)	All-cause death HR (95% CI)	CV events HR (95% CI)
Age (year)	68.2 ± 12.4	1.1 (1.0, 1.1)	1.0 (1.0, 1.0)
Male (%)	1,095 (64.8)	0.9 (0.7, 1.1)	1.0 (0.8, 1.2)
BMI (kg/m^2^)	25.1 ± 4.1	1.0 (0.9, 1.0)	1.0 (1.0, 1.0)
Diabetes mellitus (%)	554 (32.8)	1.3 (1.0, 1.6)	1.1 (0.9, 1.4)
Hypertension (%)	1,162 (68.7)	1.5 (1.2, 1.9)	1.2 (1.0, 1.5)
Hyperlipidemia (%)	350 (20.7)	0.7 (0.5, 0.9)	0.9 (0.7, 1.1)
Current smoker (%)	544 (32.2)	0.9 (0.7, 1.1)	0.9 (0.7, 1.1)
Coronary heart disease (%)	1,323 (78.2)	0.8 (0.6, 1.1)	0.9 (0.7, 1.1)
Atrial fibrillation (%)	296 (17.5)	1.7 (1.3, 2.2)	1.4 (1.1, 1.9)
Previous stroke (%)	207 (12.2)	2.1 (1.5, 2.8)	1.7 (1.2, 2.3)
COPD (%)	209 (12.4)	2.3 (1.7, 3.1)	1.3 (1.0, 1.7)
Renal insufficiency (%)	407 (24.1)	2.2 (1.7, 2.8)	1.2 (0.9, 1.5)
Creatinine level ≥ 106 µmol/L (%)	536 (31.7)	2.7 (2.2, 3.4)	1.6 (1.3, 2.0)
NYHA functional class [*n* (%)]			
II	719 (42.5)	1	1
III	618 (36.5)	1.6 (1.2, 2.0)	0.9 (0.8, 1.2)
IV	354 (20.9)	2.1 (1.6, 2.8)	1.3 (1.0, 1.7)
Respirator (%)	47 (2.8)	3.5 (1.9, 6.3)	1.7 (0.9, 3.3)
Electrocardiogram monitoring (%)	1,158 (68.5)	0.8 (0.6, 1.0)	1.2 (1.0, 1.5)
PCI (%)	565 (33.4)	0.3 (0.2, 0.4)	0.7 (0.6, 0.9)

BMI, body mass index; COPD, chronic obstructive pulmonary disease; NYHA, New York Heart Association; PCI, percutaneous coronary intervention; CV event, cardiovascular event (cardiovascular death or heart failure readmission).

Values are mean ± SD or %.

Table 2 shows the patient profiles before and after propensity score matching. Before propensity score matching, the patients in the MI group were more likely to be male (*P* < 0.001), to be current smokers (*P* < 0.001), and to have coronary heart disease (*P* < 0.001), electrocardiogram monitoring (*P* < 0.001), PCI (*P* < 0.001), and higher BMI values (*P* < 0.001). Compared with the MI group, the non-MI group had higher rates of atrial fibrillation (*P* < 0.001), COPD (*P* = 0.003), renal insufficiency (*P* < 0.001), and creatinine ≥106 µmol/L (*P* < 0.001), NYHA class III (*P* < 0.001), and NYHA class IV (*P* < 0.001). The two groups had patients with similar ages (68.6 ± 11.4 and 67.8 ± 13.3 with and without myocardial infarction, respectively, *P* = 0.164) and comparable rates of diabetes (*P* = 0.097), hypertension (*P* = 0.991), and hyperlipidemia (*P* = 0.997). 0.782), previous stroke (*P* = 0.898), and ventilator use (*P* = 0.064). Of the 439 matched pairs obtained after the first match, 290 in the MI group underwent PCI. However, the 308 matched pairs obtained after adding PCI for the second time to the matching group excluded all patients who underwent PCI. After the first and second propensity score matching, the two groups were well matched on parameters.

**Table 2:** Baseline characteristics before and after propensity score matching.

Characteristic	Before PSM			After PSM (PCI is not added to match)			After PSM (Added PCI to match)		
Variable	MI (*N* = 873)	Non-MI (*N* = 818)	*P*-value	MI (*N* = 439)	Non-MI (*N* = 439)	*P*-value	MI (*N* = 308)	Non-MI (*N* = 308)	*P*-value
Age (year)	68.6 ± 11.4	67.8 ± 13.3	0.164	69.72 ± 11.55	71.12 ± 10.77	0.063	71.19 ± 11.27	71.17 ± 10.58	0.982
Male (%)	608 (69.6)	487 (59.5)	<0.001	282 (64.2)	262 (59.7)	0.186	201 (65.3)	186 (60.4)	0.243
BMI (kg/m²)	25.5 ± 4.1	24.7 ± 4.1	<0.001	25.47 ± 4.04	24.93 ± 4.08	0.048	25.15 ± 3.97	24.89 ± 4.07	0.424
Diabetes mellitus (%)	302 (34.6)	252 (30.8)	0.097	159 (36.2)	164 (37.4)	0.779	105 (34.1)	114 (37)	0.500
Hypertension (%)	600 (68.7)	562 (68.7)	0.991	326 (74.3)	323 (73.6)	0.877	224 (72.7)	237 (76.9)	0.265
Hyperlipidemia (%)	183 (21.0)	167 (20.4)	0.782	96 (21.9)	95 (21.6)	1.000	56 (18.2)	66 (21.4)	0.362
Current smoker (%)	316 (36.2)	228 (27.9)	<0.001	145 (33)	127 (28.9)	0.214	105 (34.1)	91 (29.5)	0.260
Coronary heart disease (%)	866 (99.2)	457 (55.9)	<0.001	432 (98.4)	432 (98.4)	1.000	305 (99)	305 (99)	1.000
Atrial fibrillation (%)	99 (11.3)	197 (24.1)	<0.001	76 (17.3)	96 (21.9)	0.106	45 (14.6)	65 (21.1)	0.045
Previous stroke (%)	106 (12.1)	101 (12.3)	0.898	56 (12.8)	57 (13)	1.000	44 (14.3)	39 (12.7)	0.636
COPD (%)	88 (10.1)	121 (14.8)	0.003	65 (14.8)	73 (16.6)	0.516	46 (14.9)	54 (17.5)	0.444
Renal insufficiency (%)	169 (19.4)	238 (29.1)	<0.001	104 (23.7)	129 (29.4)	0.066	82 (26.6)	93 (30.2)	0.371
Creatinine level ≥ 106 µmol/L (%)	231 (26.5)	305 (37.3)	<0.001	157 (35.8)	171 (39)	0.364	118 (38.3)	124 (40.3)	0.680
NYHA functional class [*n* (%)]			<0.001			0.027			0.291
II	426 (48.8)	293 (35.8)		179 (40.8)	151 (34.4)		124 (40.3)	107 (34.7)	
III	289 (33.1)	329 (40.2)		146 (33.3)	184 (41.9)		117 (38)	121 (39.3)	
IV	158 (18.1)	196 (24.0)		114 (26)	104 (23.7)		67 (21.8)	80 (26)	
Respirator (%)	18 (2.1)	29 (3.5)	0.064	12 (2.7)	18 (4.1)	0.353	10 (3.2)	12 (3.9)	0.828
Electrocardiogram monitoring (%)	709 (81.2)	449 (54.9)	<0.001	290 (66.1)	252 (57.4)	0.010	217 (70.5)	175 (56.8)	<0.001
PCI (%)	565 (64.7)	0 (0.0)	<0.001	251 (57.1)	0 (0.0)	NA	0 (0.0)	0 (0.0)	NA

PSM, propensity score matching; MI, myocardial infarction.

Table 3 presents the risk of primary and secondary outcomes in the propensity score-matched cohort. Without adding PCI to the matched 439 pairs, the MI group had 113 all-cause deaths (12.87%) compared with 158 all-cause deaths (18.00%) in the non-MI group (HR 0.6; 95% CI 0.5–0.8; *P* < 0.001). After adding PCI to the matched 308 pairs, there were 110 all-cause deaths (17.80%) in the MI group and 107 all-cause deaths (17.80%) in the non-MI group (HR 1.0; 95% CI 0.7–1.5; *P* = 0.88). In the first PSMA, CV events occurred in 254 patients (28.93%) with MI and 263 patients (30.00%) without MI (HR 0.9; 95% CI 0.7–1.2; *P* = 0.52). In the second PSMA (with PCI matched), CV events occurred in 191 patients (31.00%) with MI and 184 patients (29.80%) without MI (HR 1.1; 95% CI 0.8–1.5; *P* = 0.71).

**Table 3:** Risk of primary and secondary outcomes in the propensity score-matched cohort.

Outcome	PCI is not added to match				Added PCI to match			
	No. of patients with event	Event rate %	Hazard ratio (95% CI)	*P*-value	No. of patients with event	Event rate %	Hazard ratio (95% CI)	*P*-value
All-cause death			0.6 (0.5, 0.8)	<0.001			1.0 (0.7, 1.5)	0.88
MI	113	12.87%			110	17.80%		
Non-MI	158	18.00%			107	17.80%		
CV events			0.9 (0.7, 1.2)	0.52			1.1 (0.8, 1.5)	0.71
MI	254	28.93%			191	31.00%		
Non-MI	263	30.00%			184	29.80%		

PCI is not added to match: The propensity score-matched cohort included 439 patients in the MI group and 439 patients in the non-MI group. Added PCI to match: The propensity score-matched cohort included 308 patients in the MI group and 308 patients in the non-MI group.

**Figure 2:**
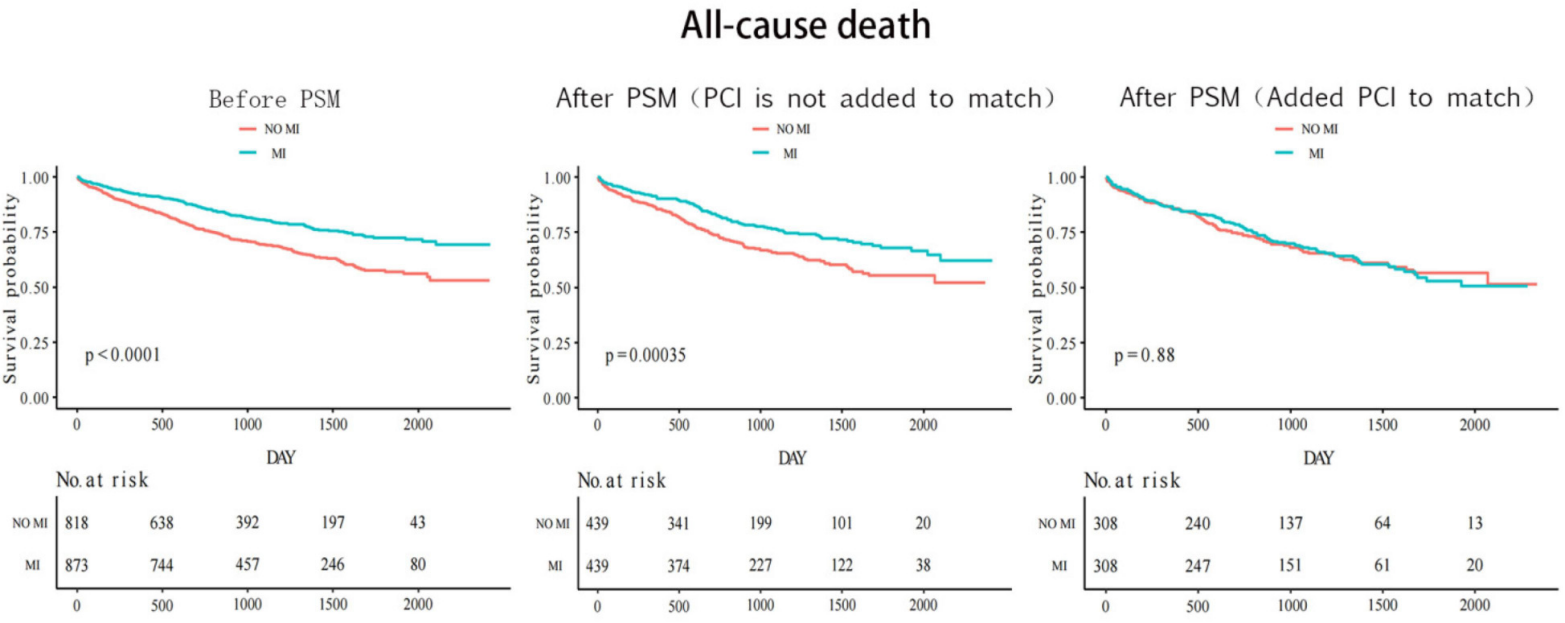
Kaplan–Meier curves of all-cause mortality before and after twice PSM matching.

**Figure 3:**
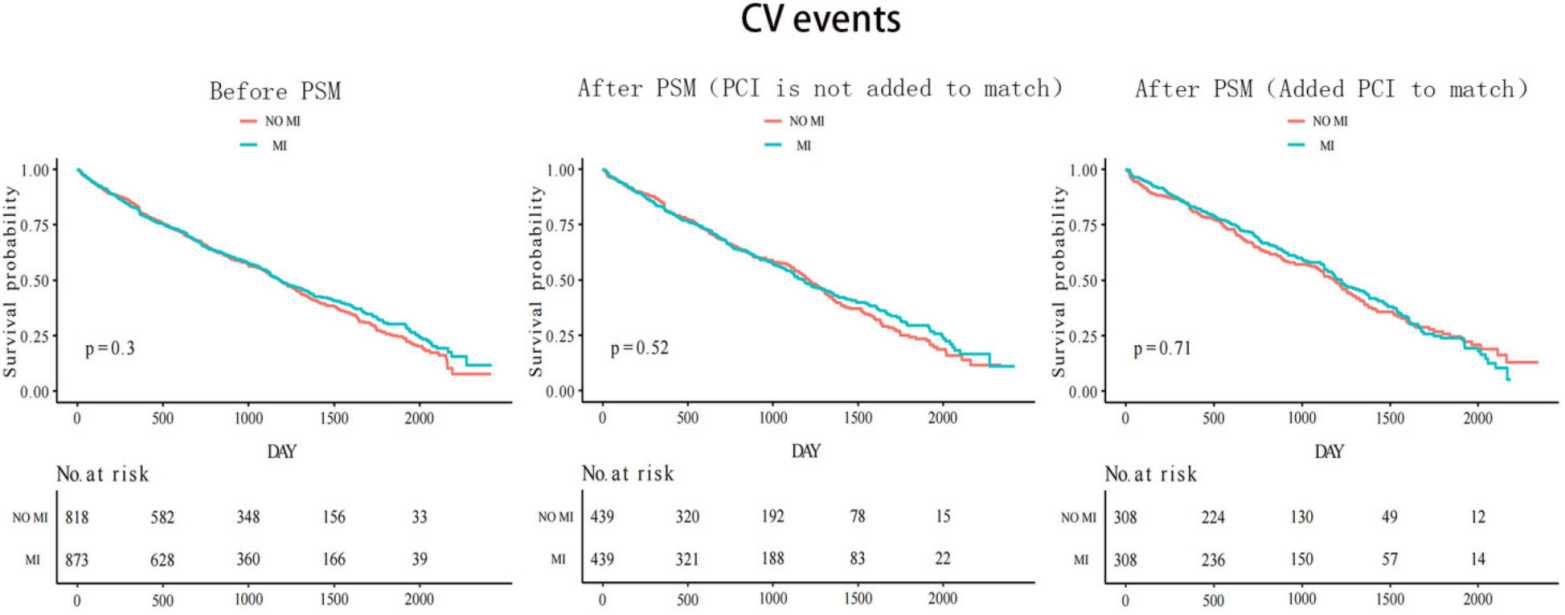
Kaplan–Meier curves of CV events before and after 2 times PSM matching.

The median follow-up time was 33 months for both groups with and without myocardial infarction. Figure 2 shows that before the propensity score match, the Kaplan–Meier cumulative all-cause mortality was lower in the MI group than that in the non-MI group (*P* < 0.0001). After the first propensity score matching, the MI group still had lower all-cause mortality than that of the non-MI group (*P* = 0.00035). However, after the second addition of PCI for matching, all-cause mortality was similar in both groups (*P* = 0.88). CV events were similar between the two groups before and after matching. Before matching, the results revealed a statistically significant value of *P* = 0.3, whereas after the first matching, the *P*-value was 0.52, and after the second matching, it was 0.71 (Figure 3).

## Discussion

There were three primary outcomes determined from our study. Firstly, HFmrEF without a diagnosis of MI had higher all-cause mortality than HFmrEF patients with MI after adjusting for the first propensity score. The second finding suggested that after adding PCI to the second propensity score, the rates of all-cause death and CV events were similar in patients with and without MI with HFmrEF. Lastly, patients with HFmrEF post-MI who underwent PCI had a lower risk of all-cause mortality compared with patients with HFmrEF without MI and those with HFmrEF post-MI without PCI.

Although several studies have reported data on post-MI heart failure in recent decades (8, 9), few have directly compared post-MI HF with non-post-MI HF. For example, a study of 1,260 MI patients undergoing PCI showed that although patients with HFmrEF after MI had similar baseline characteristics, their hospitalization rates, long-term mortality, and heart failure rehospitalization differed from those of patients with HFrEF and HFpEF (10). Other studies have shown that after acute MI, the predominant HF subtypes are HFmrEF and HFpEF rather than HFrEF (11). Our cohort specifically compared HFmrEF patients with and without MI, thereby addressing a gap in the existing literature.

In the present study, PCI emerged as a strong protective factor in discharged patients with HFmrEF. Loss of cardiac function after MI remains a leading cause of morbidity in developed countries (12), and early revascularization is the only therapy shown to reduce mortality in post-MI HF with cardiogenic shock (13). PCI enables rapid relief of acute thrombotic occlusion, treatment of underlying atherosclerotic and thrombotic risk, attenuation of adverse ventricular remodeling (14), and reduction of arrhythmias. Evidence from the EPICOR study involving 11,931 ACS patients demonstrated that higher in-hospital coronary revascularization rates were independently associated with lower adjusted 2-year mortality (15). Similarly, Núñez-Gil et al. (16) found that mild HF after MI was associated with poor prognosis and increased short-term mortality, supporting the use of aggressive strategies including early catheterization and revascularization. These observations are consistent with our conclusion that PCI plays a protective role in HFmrEF.

Previous studies have reported variations in LVEF among patients with post-MI HF. Kamon et al. (17) found that HF with non-reduced EF was the predominant subtype after AMI. Alkhalil et al. (18) showed that HFmrEF after STEMI carried higher risks of death, HF hospitalization, and ventricular arrhythmias than preserved EF. Other studies have also documented distinct characteristics and prognosis for HFmrEF after MI compared with HFrEF and HFpEF (19, 20). Most research has examined either MI or HF in isolation, whereas our study directly compared HFmrEF with and without MI, finding that the prognostic differences are largely mediated by PCI use.

This study has several limitations. First, although propensity score matching was applied to reduce selection bias, the retrospective design cannot exclude unmeasured confounding. Second, the study population was derived from a single heart center in China, limiting generalizability. Third, data on the long-term use of statins, renin–angiotensin system blockers, and beta-blockers were unavailable, preventing assessment of their potential effects on morbidity and mortality. Fourth, PCI could only be assessed within the MI subgroup, as patients without MI by definition did not undergo PCI. This limits the interpretation of PCI's protective effect across the entire HFmrEF population. Fifth, although PSM balanced baseline covariates, the reduced sample size and event counts after matching may have decreased statistical power, especially in the secondary PSM analyses. Finally, data collection was based on medical records and follow-up interviews, which may be subject to reporting inaccuracies or incomplete documentation.

In conclusion, there were no differences in all-cause mortality and CV events in patients with HFmrEF with or without MI after accounting for the second propensity score matching analysis. When PCI status was included in the PSMA, patients with HFmrEF after myocardial infarction who underwent PCI had a lower risk of all-cause mortality compared with those with HFmrEF without myocardial infarction and those with HFmrEF after myocardial infarction without PCI. While no significant difference in CV events was observed, most patients with post-MI heart failure are those with preserved and mildly reduced EF. Therefore, early blood reperfusion is recommended to reduce the long-term mortality of heart failure after myocardial infarction.

This work represents an advance in biomedical science because we determined that PCI is a protective factor for all-cause death, while no significant difference in CV events was observed. Most patients with post-MI heart failure are those with preserved and mildly reduced EF.

## Data Availability

The raw data supporting the conclusions of this article will be made available by the authors, without undue reservation.
